# Effect of a parenting education program on girls’ life satisfaction in governmental guidance schools of Shiraz

**Published:** 2014-01

**Authors:** MOHAMMAD HOSSIEN KAVEH, LEILA MORADI, LEILA GHAHREMANI, HAMID REZA TABATABAEE

**Affiliations:** 1 Department of Health Education and Promotion, Faculty of Health, Research Center for Health Sciences, Shiraz University of Medical Sciences, Shiraz, Iran;; 2 Department of Health Education and Promotion, Faculty of Health, Shiraz University of Medical Sciences, Shiraz, Iran;; 3 Department of Epidemiology, Faculty of Health, Shiraz University of Medical Sciences, Shiraz, Iran

**Keywords:** Adolescent, Life, Satisfaction, Education

## Abstract

**Introduction**: One of the main determinants of adolescents’ life satisfaction is parenting skills. Due to the lack of educational trials in this field, this research was done to evaluate the effect of a parenting education program on girls’ life satisfaction in governmental guidance schools of Shiraz.

**Methods**: This study is an educational randomized controlled trial. At first, 152 female students in 2nd grade of governmental guidance schools and 304 parents (152 mother and 152 father) were selected by multistage random cluster sampling method. Then, they were categorized into experimental and control groups. Before and after the intervention, data were collected from two groups using multidimensional students’ life satisfaction scale with stability (Cronbach's alpha=0.89), test–retest and correlation coefficient (r=0.70). Educational intervention for parents was performed in the experimental group through presentations with question and answer, discussion in small groups and distribution of educational booklets in 5 volumes. Finally, the data were analyzed using SPSS 14 and through Mann-Whitney test, Chi-square test, Fisher’s Exact test, Wilcoxon test.

**Results**: Before the intervention, the experimental and control groups did not show a statistically significant difference based on the demographic variables. Thetotal of life satisfaction scores and also its subscales in the experimental and controlgroup, before and six weeks afterthe educational interventiondid showstatisticallysignificant difference (p<0.001). The scores of differences (pre-test/post-test) in total life satisfaction between the experimental and control groups were statistically significant difference (p<0.001).

**Conclusion**: According to low scores of the students in the pre-test, especially in the control group which didn’t undergo any educational program, holding scheduled educational intervention is necessary. This study not only supports the effectiveness of educational intervention but also recommends further educational research to develop knowledge regarding patterns of parenting education.

## Introduction

Life satisfaction is one of the basic predictors of mental health and a key index of subjective well-being (1). Subjective well-being has two subdivisions: cognitive and emotional. Cognitive subdivision usually refers to life satisfaction (2). Life satisfaction is attitude and total evaluation which everybody has towards his/her quality of life or other aspects of his/her life like family, friends, and community (1). In other words, life satisfaction is an arbitration process which evaluates the quality of life in comparison with individual’s standards in person. The closer quality of life to standards is the higher life satisfaction (3). Life satisfaction in adolescence, which is a critical period, has an important role in health and individual’s quality of life in adulthood (4).

According to evidence, high life satisfaction in adolescents is accompanied with self-esteem (5) self-efficacy (3), scientific success (6, 7)and coping with stress (8).

Until now researchers have expressed that many factors like family, school, friends and community are important in the feeling of life satisfaction in adolescents, among which family has the most important role in the feeling of life satisfaction (9). Studies have confirmed that such family characteristics such as interaction between parents and their children, specifically parenting skills, is an effective key on well-being and mental health of children. So enhancement of parenting skills and appropriate relationship with children is a priority which should be noticed (10, 11). 

Disputes between parents and adolescents or poor performance of family can cause low life satisfaction, poor academic performance, behavioral problems and substance abuse (12).

The parenting in early adolescence has an effective role to prevent risky behaviors in adolescents (13). Altogether, studies’ findings have shown that parental education is an effective intervention. Educational programs for adolescents’ parents can enhance self-efficacy and parenting skills of parents, helping to reduce behavioral problems and risk among adolescents and enhance their mental health and well-being (14). While effectiveness of parenting programs in reducing behavioral problems of children is obvious, few parenting programs have been done to prevent behavioral problems in adolescents (4, 15). Many parenting interventions have been held for children’s parents and aren’t expected to prevent adolescence problems. Adolescence is accompanied with a range of social and evolutionary challenges which do not exist in childhood (16). Evidence has shown that families don’t have enough knowledge or competency for parenting, and describe it as a harsh and stressful period (15). The purpose of this study was to determine the effect of parenting education program on life satisfaction of female students in governmental guidance schools of Shiraz city during 2012-2013 school years. Our assumptions in this study indicate level of life satisfaction and its subscales (family, friends, school, self, living environment) in parents of experimental group after educational interventionis different in comparison with parents in control group.

## Methods

This study is an educational randomized controlled trial. Research’s samples have been selected among girl students of governmental guidance school in 1 and 4 educational regions of Shiraz city and also their parents’. The reasons of sample selection in the present study was availability to students and their parents and school’s environment play an important role in facilitating planning, implementation and evaluation educational intervention. 152 female students in the second grade of guidance school and also 304 parents (152 mother and 152 father) were the study sample. Sampling was done through multistage random cluster method. In the first phase, 4 educational districts in Shiraz were randomly categorized into two categories (1 and 2 in the same category, 3 and 4 in the other category) and then one district was randomly selected from each category. Then, two schools were selected randomly from each district with criterion to have at least 2 classes in the second grade of guidance school. Selected schools in each area were divided into experimental and control groups randomly. In the next stage, second-grade classes were selected; if second-grade classes in each school were more than the required classes, it was selected randomly. Finally, students’ parents who had at least elementary school education participated in the study after signing written consent. The number of students in the experimental and control groups was 80 and 72 respectively and that of their parents in the experimental and control groups was 160 and 144, respectively. Multidimensional students’ life satisfaction scale (Huebner 1998) was used to evaluate life satisfaction in students (17). Stability of this instrument was confirmed by Griffin, Huebner’s and Zaki studies and reliability coefficient was reported %70 to %90 in two and four week intervals by Cronbach's alpha and Test–retest method (18-20). MSLSS is a self-report scale with 40 statements which measures student’s satisfaction in different aspects of life and includes 5 subscales: family (7 statements), friends (9 statements), school (8 statements), environment (9 statements) and self (7 statements). Students’ response to each statement (for example, my parents treat me fairly) is scored 1 to 6 on Likert scale (completely agree, agree, slightly agree, slightly disagree, disagree, completely disagree) and in total, minimum and maximum of acceptable score were 40 and 240, respectively. Pretest was done for the students of the experimental and control groups, using MSLSS scale (17). Afterwards, educational intervention for parents of the experimental group was held in 5 sessions (each session 120 minutes) in which active methods such as interactive lecture accompanied with question and answer, discussion and teamwork in small groups and educational video clips were used. In addition, an educational booklet in 5 volumes was distributed among the parents. Also, educational short messages were sent by mobile phone to repeat the contents of the previous session and enhance the parents’ learning.

Educational subjects which were explained and discussed are as follows:

- Understanding adolescence and its social, cultural and educational harms.
- Family role, especially parents in health and adolescent satisfaction.
- Analysis of hopes and parents’ challenges in upbringing of children.
- Identification and analysis of challenges and required skills in parenting.
- Parents’ familiarity to effective communication to their children.

Acquisition of knowledge regarding basic skills (for example: assertiveness skills) to handle educational issues of children.

After six weeks of educational intervention, post-test was done for the students of the two groups (experimental and control) using MSLSS scale ( 17 ).

On the other hand, after training, a self–assessment questionnaire was used to assess the effect of educational program on parents’ learning in the experimental group. This researcher- made questionnaire included 10 key subjects of educational content (for instance, my knowledge regarding methods to strengthen relationship with adolescents before and after attending the educational course). Respondents evaluated their knowledge for each subject on a five-degree scale of Likert type (very much, much, average, little, very little) and reported it before and after training. Each item was scored 5 for highest and 1 for lowest. Finally, knowledge scores ranged from 10 to 50. Educational process and also participants’ satisfaction about it in the experimental group was assessed by 13 questions (for example, in educational course of parenting skills, how much was discussion in small groups useful and applicable?) and on a five-point scale of Likert type (very much, much, average, little, very little) Assessment scores of education process and parents’ satisfaction, specifically for present study were categorized, the contract in three levels. People whose assessment scores were less than 32.1 were categorized in below the desirable level, between 32.1 to 45 in the desirable level, and between 45.1 to 65 in higher than the desirable level.

Research data were analyzed in SPSS 14 (SPSS Inc, Chicago, IL, USA) using statistical tests such as Mann-Whitney test, Chi-square, Fisher’s Exact test, Wilcoxon test. 

## Results

The students’ mean age in the experimental and control groups were 12.84±0.60 and 12.76±0.66,respectively.Mann-Whitney test did not show a statistically significant difference between the experimental and control groups based on the age (p=0.419) or other demographic variables studied (Table 1).

**Table 1:** Comparison of the experimental and control groups based on the demographic variables

**Groups** **Variables**	**Control group (n=72)**		**Experimental group (n=80)**		**Mann Whitney test**
	**Range**	**Mean±SD**	**Range**	**Mean±SD**	**p**
Students age	12-14	12.76±0.66	12-14	12.84±0.60	0.419
Fathers age	33-57	44.03±5.76	34-55	43.96±4.87	0.906
Mothers age	28-52	37.64±4.83	28-52	37.99±5.28	0.567
Size family	3-7	4.38±0.90	3-9	4.40±0.94	0.624
Number of children	1-5	2.38±0.89	1-7	2.40±0.94	0.624
Mean of family income in month (Tomans)	3 -20 million	733472±780911.63	3 million,500 Thousand- 20 million	685000±269598.86	0.578

The percentage of fathers in the experimental and control groups and mothers in the two groups which had high school and diploma education were 47.5%, 47.3%, 55% and 59.7% respectively and the percentage of fathers in experimental and control groups which had elementary education were 15% and 13.9% respectively too. And also mothers’ educations which was related to academic education in the experimental and control groups were 6.3% and 11.1%, respectively. Chi-Square test didn’t show a significant difference between the experimental and control groups in terms of fathers and mothers’ education (Fathers: p=0.985, Mothers: p=0.332).

The percentage of fathers in the experimental and control groups who were self employed were 55% and 51.4% respectively, and the percentage of them in the two groups who were unemployed were 1.3% and 1.4% respectively; the remaining were employed, worker and retired. Chi-Square test didn’t show a significant difference between the study groups in terms of the fathers’ job (p=0.959).

As to mothers’ job, the percentage of mothers in the experimental and control groups who were housewives were 98.8% and 98.6% respectively and the rest of them who were employed in the two groups were 1.3% and 1.4% respectively. Fisher’s Exact test didn’t show a significant difference between the study groups regarding the mothers’ job (p=0.725).

After educational intervention in the experimental group, median of total life satisfaction scores significantly increased to 191.5 (Wilcoxon test, p<0.001); a moderate decline of 14 scores was observed in the post-test of students’ median of total life satisfaction scores in the control group (Median=159). This decline was significant in terms of Wilcoxon test (p<0.001). Comparison of the values of pre and post-test for each subscale of life satisfaction showed that life satisfaction scores in the experimental group increased significantly in all subscales of life satisfaction (p<0.001), but in the control group which didn’t have parenting education program a statistically significant decline was observed in all subscales of life satisfaction scores (p<0.001) (Table 2).

**Table 2:** Comparison of life satisfaction mean scores between experimental and control group

**Groups** **Life satisfaction and subscales**		**Control group (n=72)**					**Experimental group (n=80) **			
		**Med**	** Score range**			**Wilcoxon signed ranks test**	**Med**	**Score range**		**Wilcoxon signed ranks test**
			**Lower**		**Upper**	**p**		**Lower**	**Upper**	**p**
**Friends satisfaction**	Pre-test	37	12	54		p<0.001	36	9	47	p<0.001
	Post-test	35.5	9	52			43	28	53	
**Self satisfaction**	Pre-test	34	18	42		p<0.001	31	17	40	p<0.001
	Post-test	31.5	18	42			36	24	42	
**School satisfaction**	Pre-test	33	16	47		p<0.001	33	12	48	p<0.001
	Post-test	30	11	45			39	14	48	
**Family satisfaction**	Pre-test	35	9	42		p<0.001	29	11	42	p<0.001
	Post-test	32	7	42			37	21	42	
**Living environment satisfaction**	Pre-test	36	13	50		p<0.001	34	14	52	p<0.001
	Post-test	32	13	50			39.5	21	54	
**Total life satisfaction**	Pre-test	173	108	227		p<0.001	160	86	202	p<0.001
	Post- test	159	91	211			191.5	148	232	
										

Mean scores of the differences in total life satisfaction (post test-pre test) in the experimental and control groups were 32.06±17.26 and -12.55±13.29, respectively; there was a significant difference between them based on Mann-Whitney test (p<0.001). In the experimental group, the lowest increase was related to self- satisfaction which was 4.95±4.20 and the greatest increase is related to friends’ satisfaction which was 7.95±5.86. In the control group, the minimal decline was seen in self-satisfaction which was-1.69±3.36 and the greatest decrease was in friends’ satisfaction which was -3.11±5.20 (Table 3).

**Table 3:** Comparison of the mean differences scores of life satisfaction* between experimental and control groups

**Groups** **Life satisfaction ** **and subscales**	**Control group (n=72)** **(post-pre)**	**Experimental group (n=80) (post-pre)**	** Mann- whitney test**
	**Mean±SD**	**Mean±SD**	**p**
**Friends satisfaction**	-3.11±5.20	7.95±5.86	p<0.001
**Self satisfaction**	-1.69±3.36	4.95±4.20	p<0.001
**School satisfaction**	-1.72±4.76	6.21±6.03	p<0.001
**Family satisfaction**	-3.01±4.31	6.87±4.88	p<0.001
**Living environment satisfaction**	-3.01±5.04	6.07±5.09	p<0.001
**Total life satisfaction**	-12.55±13.29	32.06±17.26	p<0.001

* Mean difference scores of life satisfaction was calculated by: post-test (6 week after program) – pre-test

Before the intervention, the parents’ median scores of knowledge in the experimental group was29 and after the intervention it was 40.Wilcoxon test did show a significant difference in parents statistically (p<0.001) (Table 4).

**Table 4:** Comparison of the mean scores of knowledge in parents, after and before intervention in experimental group

**Experimental group**							
**Parents**	**Pre-program**			**6 week after program**			**Wilcoxon**
**Parents knowledge**	**Med**	**Score range**		**Med**	**Score range**		**p**
		**Lower**	**Upper**		**Lower**	**Upper**	
**Mother** **(n=80)**	29	10	42	41	19	50	p<0.001
**Father** **(n=80)**	29	10	40	40	21	50	p<0.001
**Total**	29	10	42	40	19	50	p<0.001

After the intervention, mean and median of assessment scores on education process and parents’ satisfaction in the experimental group were 49.59±7.175 and 50, respectively. The median shows that at least half of the parents have evaluated the quality of the program as highly desirable. In assessing the education process and parents’ satisfaction, scores ranged from 13 to 65. In this assessment, 116(%72.5) parents reported the education process as highly desirable, 40(%25) asdesirable and 4(2.5%) less than desirable level. Chi–square test showed a significant difference between parents’ satisfaction and education process (p<0.001). 

## Discussion

In this study, the mean scores of total life satisfaction and its subscales (friend’s satisfaction, self-satisfaction, school satisfaction, family satisfaction and living environment satisfaction) in female students of the experimental group compared with the control group increased after six weeks of educational intervention, while the mean scores of female students in the control group decreased six weeks after the educational intervention. This finding confirms the effectiveness of educational intervention of parenting skills for the experimental group’s parents in increasing the total life satisfaction and its subscales of their students.

In our research, evidence of similar studies on the impact of parenting education program on life satisfaction of adolescents was not found, but Shannon and et al. (2004) (21), Milevsky (2007) (22) Saha (2010) (23) and Cenkseven (2012) (24) have confirmed that there is a significant relationship between parenting styles and quality of family relationships with adolescents’ life satisfaction.

Several studies have shown that parents’ support (25, 26) and quality of family relationship (27) have an important role in causing bond feeling and also positive relationship with family, school, friends, living environment and oneself and that through interventional programs we can improve the positive relationship in adolescents (28, 29) Relationships between individuals are the main element to form adolescents’ life satisfaction. Positive relationship with family and friends, school and living environment are the main protective factor in preventing behavioral problems and causing well-being and adolescents’ satisfaction (30, 31).

The results of this study showed that total life satisfaction and its subscales (friends’ satisfaction, self-satisfaction, school satisfaction, family satisfaction and living environment satisfaction) in the control group’s students decreased; this was similar to previous studies’ findings. Based on the existing evidence, in the beginning and progression of adolescence, total life satisfaction, family satisfaction, friends’ satisfaction, self-satisfaction, school satisfaction and living environment satisfaction are decreased. Decreased life satisfaction in adolescence has been considered as a natural evolutionary phenomenon. Adolescents are faced with various challenges during transition of childhood to adolescence which can cause a decline in life satisfaction (32-35).

Reduction in adolescents’ positive relationships is accompanied with life dissatisfaction and these are related to several negative outcomes such as low self-esteem (36) problems of befriending risk of temptation by friends (37), poor academic performance (27) depression (7) anxiety, suicidal thoughts (38), and substance abuse (39) Based on this evidence, it is suggested that the quality of interpersonal communications should improve as a solution to enhance life satisfaction and prevent harms in adolescents. Effectiveness of parenting educational programs to improve relationships (19) and enhance parental competencies has been confirmed by numerous studies (40). In this study also educational programs held for parents on subjects like special focus on relationships between parents and adolescents, providing information and strategies for parents to hold and improve positive relationship with adolescents and how to confront with behavioral problems of adolescents, and parents’ special attention to learn communication skills and adolescents’ assertiveness caused family satisfaction, friends satisfaction, self-satisfaction, school satisfaction and living environment satisfaction in students. During the educational intervention, interactive methods, discussion in small groups and providing opportunities to ask and answer questions during and after the educational sessions were used. Group discussion is the most useful and the most prestigious educational methods and among them discussion is mostly used. Effectiveness of using group discussion in parent education has also been confirmed in other studies.

For instance, the findings of Barlowet al.’s (2000)study showed that group discussion had a great impact, facilitates better knowledge retention (41), causing positive changes on parents’ attitude and behavior and also in enhancing parenting skills (41).

Lack of a significant difference between experimental and control groups in relation to demographic variables is worth considering; educational intervention designed and implemented in this study for parents had been appropriate for research community despite demographic differences. In other words, educational method and educational content which were used for parents in schools can be useful in community. On the other hand, the results of the post-test showed an increase of 11 scores in parents’ knowledge after the intervention which indicates that implementation of educational program has responded to educational needs of parents significantly and parents’ high level of satisfaction of education can confirm the program’s efficacy.

## Conclusion

According to the effectiveness of the present study and lack of enough knowledge of parents regarding parenting, designing educational interventions based on interactive teaching methods and appropriate themes for adolescents’ parents is recommended. Also further educational trials are suggested by using various methods and theories. 

**Limitations & Suggestions**

This study has been done in governmental schools. However, schools have been selected randomly and they may not be completely generalizable to other geographical areas because of cultural, social and economical differences.Moreover, using the study findings cannot be generalized about other interventional educational methods such as electronic learning or a combination of educational and consulting intervention. Finally,in this study only parental education’s effect on the female adolescents has been evaluated. In order to promote life satisfaction in adolescents and also parenting skills can be suggested by researchers to design and implement educational trials to improve parenting skills for parents who have male adolescents with concurrent participation of fathers and mothers in 4 educational regions. In addition, comparing researches for parents of two sexes can be done. Also long-term effects of parenting education program should be assessed. On the other hand teachers and school counselors’ role would be considered regarding all aspects ofadolescents’life satisfaction like school-satisfaction, self-satisfaction, friends-satisfaction and educational trials for them should be designed and implemented.

**Ethical consideration**

Educational administrators gave authorization before research implementation. After describe explanations, participants signed written consent which were included introduce about the study, objectives an implementation method. In addition, participants were assured questionnaires’ information remain secret and doesn’t have name to identify and information the collectively be analyzed and reported. Meanwhile it’s been announced voluntary to participate in the study. After research implementation, some of educational contents which were main points have been offered to control group for regard of ethical consideration.

**Implications**

Findings of this study can be used where training and parenting consultation are done, e.g.schools, educational centers, family consultation centers and media which can be useful and usable. 
